# A genome scan for quantitative trait loci affecting growth-related traits in an F1 family of Asian seabass (*Lates calcarifer*)

**DOI:** 10.1186/1471-2164-7-274

**Published:** 2006-10-26

**Authors:** Chun Ming Wang, Loong Chueng Lo, Ze Yuan Zhu, Gen Hua Yue

**Affiliations:** 1 Molecular Population Genetics Group, Temasek Life Sciences Laboratory, 1 Research Link, National University of Singapore, 117604, Singapore

## Abstract

**Background:**

Body weight and length are economically important traits in foodfish species influenced by quantitative trait loci (QTL) and environmental factors. It is usually difficult to dissect the genetic and environmental effects. Asian seabass (*Lates calcarifer*) is an important marine foodfish species with a compact genome (~700 Mb). The recent construction of a first generation linkage map of Asian seabass with 240 microsatellites provides a good opportunity to determine the number and position of QTL, and the magnitude of QTL effects with a genome scan.

**Results:**

We conducted a genome scan for QTL affecting body weight, standard length and condition factors in an F1 family containing 380 full-sib individuals from a breeding stock by using 97 microsatellites evenly covering 24 chromosomes. Interval mapping and multiple QTL model mapping detected five significant and 27 suggestive QTL on ten linkage groups (LGs). Among the five significant QTL detected, three (*qBW2-a*, *qTL2-a *and *qSL2-a*) controlling body weight, total and standard length respectively, were mapped on the same region near *Lca287 *on LG2, and explained 28.8, 58.9 and 59.7% of the phenotypic variance. The other two QTL affecting body weight, *qBW2-b *and *qBW3*, were located on LG2 and 3, and accounted for 6.4 and 8.8% of the phenotypic variance. Suggestive QTL associated with condition factors are located on six different LGs.

**Conclusion:**

This study presents the first example of QTL detection for growth-related traits in an F1 family of a marine foodfish species. The results presented here will enable further fine-mapping of these QTL for marker-assisted selection of the Asian seabass, eventually identifying individual genes responsible for growth-related traits.

## Background

Asian seabass (*Lates calcarifer*), also called Barramundi in Australia, is an important farmed marine foodfish species which has been cultured for more than 20 years in Southeast Asian countries [1]. Current global annual production is nearly 400,000 MT according to FAO statistics in 2003, and over 90% of which originated from Taiwan, Hong Kong, Singapore and Thailand [2]. Australia is also presently becoming a major producer. The production of this species is expected to expand more rapidly in the next few years due to high market demand. Studies using allozyme and mtDNA polymorphism showed genetic diversity of the Asian seabass in Australia was low and genetic differentiation among fish from different regions was large [3,4], while studies using polymorphic microsatellites showed that both cultured and wild stocks of Asian seabass in Southeast Asia contained high allelic and gene diversity [5,6]. No detailed selective breeding program has yet been reported.

Growth-related traits such as body weight, total length, standard length and condition factor are quantitative traits influenced by a number of genes with relatively small effects, and environmental effects. These traits could be improved by phenotypic selection, but the genetic gain can be increased more rapidly with marker-assisted selection [7,8]. Genome scans using molecular markers have proved to be effective in the detection of QTL in organisms such as livestock species in experimental populations created by crossing genetically diverse strains [7]. Studies on linkage and QTL mapping in foodfish species started later than those in economically important livestock [9]. To date, linkage maps have been constructed for only 13 foodfish species including rainbow trout [10], salmon [11], tilapia [12], catfish [13], yellowtail [14], and common carp[15].

The genetic and molecular basis of growth-related traits is poorly understood in fish. Genetic studies in fish species have been limited by cost and the difficulty of raising large numbers of fish under the same conditions and the lack of linkage maps. A few QTL for growth-related traits, disease resistance and stress resistance have been detected in experimental F2 or backcross populations of some foodfish species, such as rainbow trout [16-18], salmon [19,20] and tilapia [21,22]. QTL analysis in F1 full-sib families from breeding stocks has not been reported in any fish species, although methods for detecting QTL with F1 populations in outbreed species have been developed [23-25], and have been successfully applied to identify QTL in several plant species [26,27].

Recently, we established a first generation genetic linkage map of Asian seabass using 240 microsatellite markers covering 24 linkage groups with marker interval of 6.2 and 4.7 cM in the female and male maps respectively [28], thus providing a necessary tool for a whole genome scan for QTL affecting economically important traits. In a previous study, we demonstrated that body weight at 90 day post-hatch (dph) was significantly correlated with body weight at harvest (289 dph) in Asian seabass [29]. In an association study, we detected significant association between grow-traits and polymorphism in one parvalbumin gene (*PVALB1*) [30]. In the present study, we conducted a genome scan for QTL affecting body weight, total length, standard length and condition factors at 90 dph in an F1 family containing 380 individuals from a breeding population. The objectives of this study are: 1) to test whether an F1 family from a breeding stock can be used for detecting QTL affecting growth-related traits, 2) to locate QTL on linkage groups, 3) to estimate effects of QTL and finally, 4) to understand QTL allele substitution effects. This study is an initial step towards the goal of marker-assisted selection in Asian seabass.

## Results

### Trait values

The phenotypic values of growth-related traits in the F1 family at 90 dph are presented in Table 1. The average body weight of the 380 F1 individuals was 29.4 ± 13.5 g, ranging from 6.7 to 68.2 g. Total lengths (TL) of these fish ranged from 75.4 to 169.0 mm with an average of 122.6 ± 20.1 mm, whereas the standard lengths (SL) were between 62.8 and 139.9 mm (average 101.3 ± 16.8 mm). The average condition factors (KTL and KSL) were 1.49 ± 0.12 and 2.64 ± 0.22 calculated according to TL and SL respectively (Table 1).

### Linkage map

**Figure 1:**
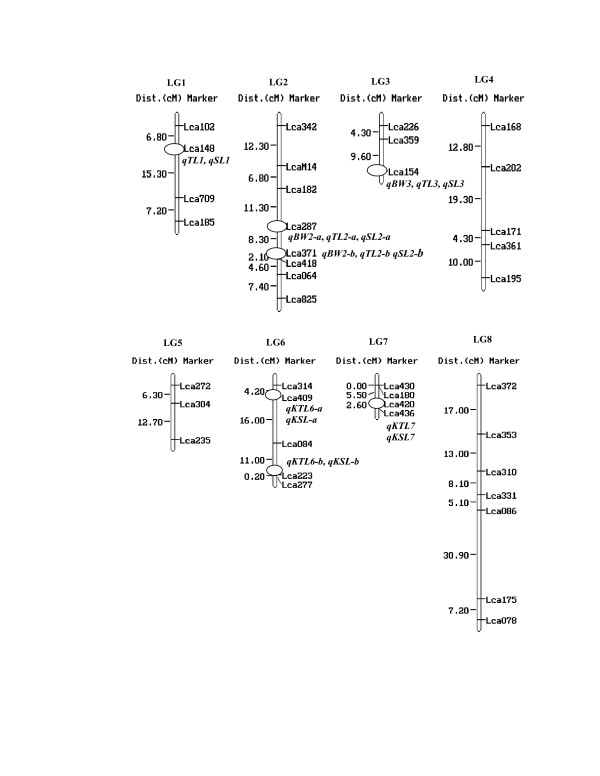
**A genetic linkage map of Asian seabass**. Linkage group (LG) 1–8. The locations of QTL affecting growth-related traits are circled on the map. The names of QTL are shown in italic and bold.

**Figure 2:**
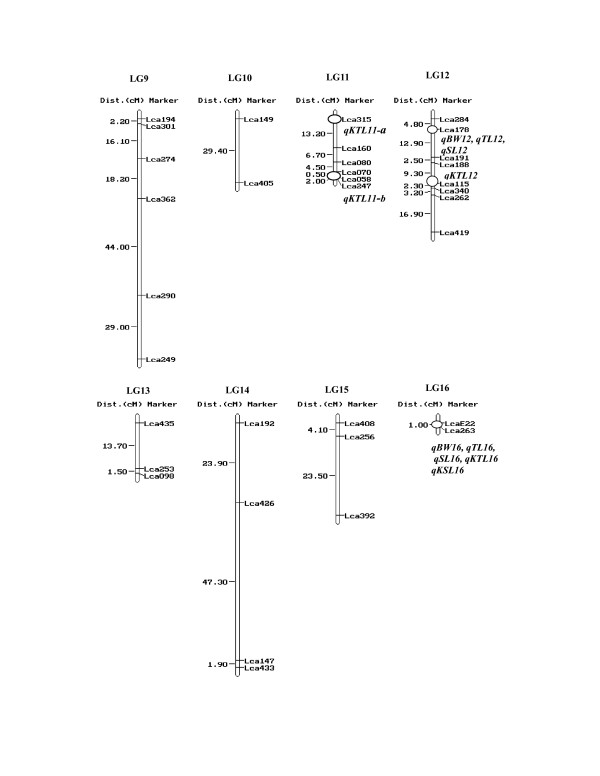
**A genetic linkage map of Asian seabass**. Linkage group (LG) 9–16. The locations of QTL affecting growth-related traits are circled on the map. The names of QTL are shown in italic and bold.

**Figure 3:**
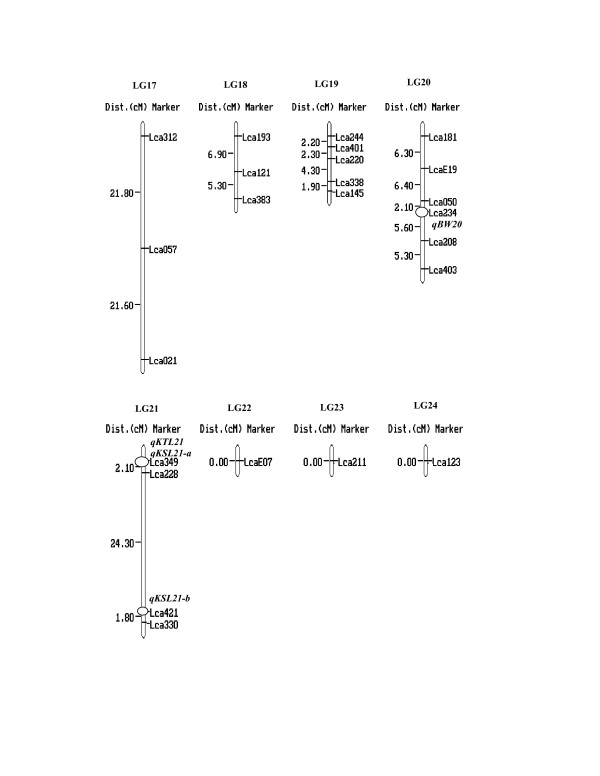
**A genetic linkage map of Asian seabass**. Linkage group 17–24. The locations of QTL affecting growth-related traits are circled on the map. The names of QTL are shown in italic and bold.

The 97 microsatellite markers selected were mapped into 24 linkage groups with a map length of 690.6 cM. 21 of the 24 linkage groups were covered with at least two markers (Figure 1, 2 and 3). The markers were well-dispersed throughout the genome, and the average distance between markers was 7.12 cM.

### QTL mapping

**Figure 4:**
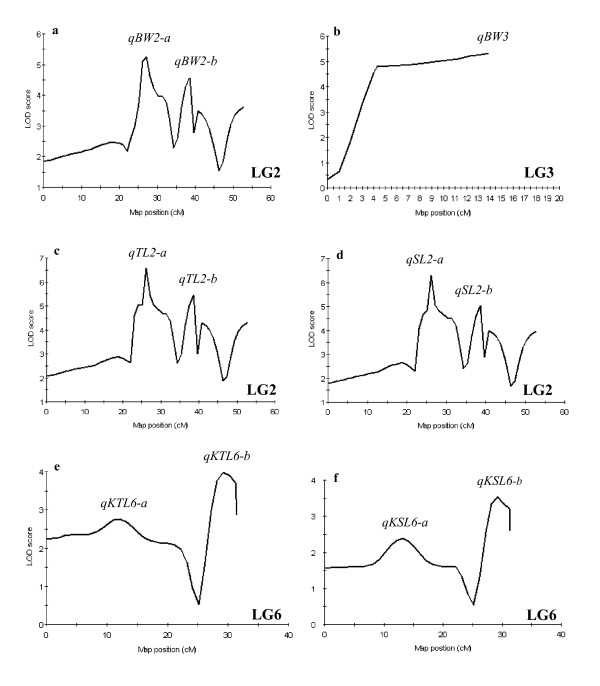
**Mapping of QTL for growth-related traits**. a, QTL for total weight on LG2; b, QTL for body weight on LG3; c, QTL for total length on LG2; d, QTL for body length on LG2; e, QTL for KTL on LG6; f, QTL for KSL on LG6. The lines were drawn by plotting the LOD scores at each marker as well as at 1.0 cM intervals along the linkage group. The QTL were detected with genome-wide threshold for a, b, c and d and linkage-group-wide threshold for e and f.

QTL affecting body weight, total length and standard length were identified on a genome-wide scale. The genome-wide LOD significance thresholds were 4.5, 6.1 and 6.1 for body weight, total length and standard length, respectively, while the linkage-group-wide LOD significance thresholds varied from 1.7 to 5.4 (Table 2). Five significant (genome-wide significant) and 27 suggestive (linkage-group-wide significant) QTL were detected by the whole genome scan (Table 2 and Figure 1, 2, 3 and 4). Multiple QTL Model (MQM) mapping with initial QTL did not change the results.

Three significant QTL (*qBW2-a*, *qTL2-a *and *qSL2-a*) controlling body weight, total length and standard length, were detected on the same region near the microsatellite marker *Lca287 *on LG2, and explained 28.8, 58.9 and 59.7% of the observed phenotypic variance, respectively (Table 2). The fourth significant QTL(*qBW2-b*) affecting body weight was detected near *Lca371 *on LG2. This QTL accounted for 6.4% of the phenotypic variance. The fifth significant QTL (*qBW3*) affecting body weight was located near *Lca154 *on LG3, and explained 8.8% of the phenotypic variance.

The 27 suggestive QTL affecting growth-related traits were located on ten linkage groups (LG1, 2, 3, 6, 7, 11, 12, 16, 20 and 21) (Table 2 and Figure 1, 2 and 3). Three suggestive QTL for body weight (i.e. *qBW12*, *qBW16 *and *qBW20*) were detected on LG12, 16 and 20, respectively. These QTL explained relatively low phenotypic variance. Six suggestive QTL for standard and total length were detected on LG1, 2, 3, 12 and 16, respectively. In the regions of *Lca148 *on LG1, *Lca287 *and *Lca371 *on LG2, *Lca154 *on LG3, *Lca436 *on LG7, *Lca178 *on LG12, *LcaE22 *on LG16, and *Lca349 *on LG21 as well, QTL controlling more than one growth-related traits were detected.

Suggestive QTL associated with the condition factors were located on six different LGs (LG6, 7, 11, 12, 16 and 21). Some suggestive QTL for KTL and KSL (i.e. *qKTL6-a *and *qKSL6-a, qKTL6-b *and *qKSL6-b, qKTL7 *and *qKSL7*; *qKTL16 *and *qKSL16, qKTL21-a *and *qKSL21-a*), were located on the same locations of LG6 7, 16 and 21, respectively, but some (e.g. *qKTL11-a, qKTL11-b, qKTL12 *and *qKSL21-a*) were mapped on different locations.

### Effects of QTL allele substitution

**Figure 5:**
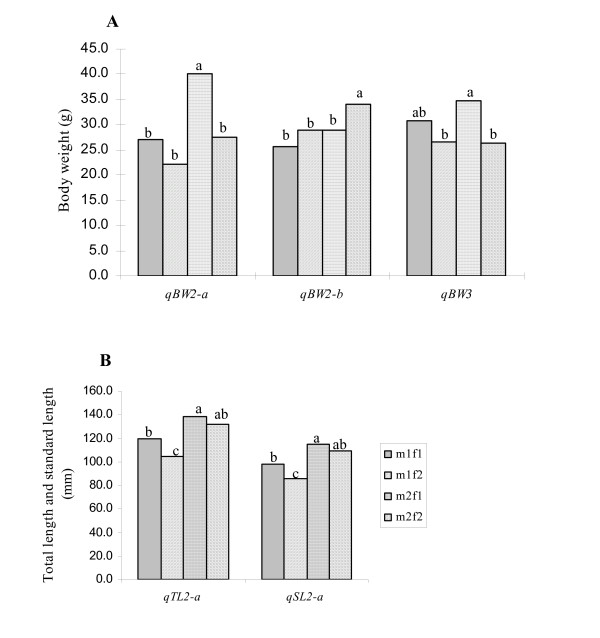
**The effects of genotypes on body weight (A), total length and standard length (B)**. The genotypes were derived from the markers near QTL peaks. Significant differences between phenotype means are the estimated effect of alternate alleles inherited from the male parent (m1 or m2), alleles inherited from the female parent (f1 or f2) and/or their interaction. The letters on the top of bars indicate the level of difference in phenotypic value between genotypes. The same letter indicates that the difference is statistically insignificant, whereas different letters represent statistically significant (Bonferroni T tests at *P *< 0.01) difference.

A two-way ANOVA was performed on the 380 progeny using four allelic combinations (m1f1, m1f2, m2f1 and m2f2) from markers nearest to the five significant QTL in order to investigate associations between phenotypic traits and genotypes. The phenotype values of each allelic combination are listed in Table 2. Out of the five significant QTL, progeny with only one m2f1 genotype at any of the four QTL, *qBW2-a*, *qTL2-a*, *qSL2-a *and *qBW3*, showed higher phenotype values than those without m2f1, while individuals with genotype m2f2 at QTL *qBW2-b *displayed heavier body weight than individuals without this genotype (Table 2 and Figure 5A and 5B). At the extreme, a difference of 15.8 g in BW were observed between individuals with genotypes m2f1 and m1f2 at the QTL *qBW2-a*. Similarly, difference in total length between individuals with genotypes m2f1 and m1f2 at QTL *qTL2-a *was 34.3 mm (Table 2 and Figure 5A and 5B). By using a single marker nearest to every suggestive and significant QTL, the effects of alleles within paternal and maternal parents and their interactions were further analyzed (Table 2). Among the five significant QTL, *qBW2-a*, *qBW2-b*, *qTL2-a *and *qSL2-a *showed significant effects within both the male and female alleles, and *qBW3 *showed significant effects within female alleles only. At suggestive QTL, QTL allele substitution effects were also observed (Table 2).

## Discussion

### Linkage mapping

As expected, the selected 97 microsatellites were mapped into 24 linkage groups. For the majority of LGs, three or more markers were mapped. Marker order in every LG were almost identical to that in our published linkage map [28]. The total map length was 690.6 cM, quite similar to the average length of male and female maps we published [28]. The average marker interval was 7.12 cM, smaller than the required minimum of 10–20 cM for QTL mapping [31]. Therefore, this linkage map is suitable for a preliminary genome-wide scan for QTL on growth-related traits.

### QTL mapping

Accurate QTL analyses have been developed in recent years to detect and estimate QTL effects on economically important livestock and plant species [32,33]. In most studies employing QTL mapping, two generations of mapping populations were created by crossing genetically different lines or strains, followed by producing second generation progeny from mating F1 males and females or back-crossing F1 progeny to its parents in order to increase the power for QTL detection [7,8,34]. However, for species such as cattle, trees and most marine fish species, long generation interval meant that creating F2 generations or backcross families will take a long time, and therefore, is not easily feasible in practice. Methods for detection of QTL in F1 (full-sib or half-sib) families were developed [23-25] and successfully applied to plants [26,27], cattle [35] and sheep [36]. The power to detect QTL depends on heritability of traits, average allelic substitution effect of the alleles involved, recombination distance between the QTL and associated markers, and sample size of the progeny [37]. In most marine fish species, as in the Asian seabass, which are broadcast spawners with a long generation interval (3–4 years), they are prolific spawners and are highly fecund. Furthermore, most cultured marine foodfish are progeny of wild-collected brooders having not been selected for any traits, as such, possess high levels of genetic variation. Hence, marine foodfish would be ideal for testing QTL analyses in F1 full-sib families. In this study we conducted a genome scan for QTL for growth-related traits in an F1 family including 380 individuals from a breeding stock. Five significant and 27 suggestive QTL were detected on the genome. Our study represents the first example of detecting QTL for growth-related traits by using a single F1 family with large number of offspring on the whole genome in marine foodfish. However, using of an F1 full-sib family from a breeding population for QTL analysis has its limitation. The power of such QTL analysis is limited. Only the QTL that are segregating in one or both parents can be detected. The detected QTL may not be representative for the genetic architecture of growth of Asian seabass. Thus, the detected QTL should be verified in other Asian seabass families before they are used in selective breeding.

Significant and suggestive QTL for body weight were detected in five LGs in this study, while in rainbow trout, QTL for body weight were detected in seven different LGs using a genome-scan [38]. The proportion of variance explained by the significant QTL for body weight varied from 6.4% to 28.8% in this study, which is similar to the 11–25% that was found in trout [38]. Among the significant QTL detected in this study, *qBW2-a*, *qTL2-a *and *qSL2-a *located on LG2, explained high percentages of the observed phenotypic variance, indicating that growth-related traits of Asian seabass might be controlled by a few QTL with large effects, together with some QTL with minor effects. Our results in this vertebrate system are consistent with a number of genetic studies in plants and insects suggesting that a large proportion of quantitative variation can be explained by the segregation of a few major QTL [39].

At some chromosomal regions, QTL controlling more than one growth-related traits were detected, suggesting either the linkage of two or more QTL (one for each trait), or the presences of a single QTL on each LG with pleiotropic effects. However, distinguishing between a single gene versus a gene cluster with individual QTL proved to be difficult, given the current marker density in the first generation linkage map[28]. More markers are needed to identify the full spectrum of genetic changes, which contribute to growth differences and to narrow down the location of a gene or genes within each chromosome interval with contribution to growth divergence. Isolation of additional microsatellites, SNPs, and microsatellites in ESTs and genes are still underway, to allow for the construction of a high resolution linkage map for fine mapping of QTL and gene of interest.

Suggestive QTL for condition (*K*) factors were located on six LGs, and their effects were relatively small. In rainbow trout, only a QTL for *K *factor was mapped in one LG [40], whereas in salmon four significant QTL for *K *factor were mapped to four LGs [19]. In Asian seabass, some QTL for the traits KTL and KSL were located on the same positions, while others were located in different places, suggesting that the two *K *factors are controlled by some genes in common and some different genes with small effects. Since the QTL were detected in only an F1 full-sib family and the effects of these QTL are small, confirmation in other families is required.

We have noticed that the total PVE of six significant and suggestive QTL for the trait standard length was almost 100%, which is theoretically impossible. However in practice, similar results have been reported in previous QTL analyses. For example, in the three published papers [19,41,42] on QTL mapping in fish, the total PVE of QTL for a certain trait was over 100%. There might be several reasons for this phenomenon. Firstly, interaction among QTL was not included in QTL analysis due to the limitation of the software. If the correlation between QTL is large, they share a certain amount of variation and are not completely independent. Secondly, the proportion of the variation explained by these QTL is likely an overestimate due to the Beavis effect [43,44]. More makers on the linkage map, larger population size and more improved statistic models to detect QTL interactions are expected to solve this problem.

A two-way ANOVA revealed that allelic substitution at the microsatellite markers located near the five significant QTL showed large effects on growth-related traits. Certain allelic combinations showed significantly improved performances of the traits analyzed. The genotypes at these marker loci may be useful for growth improvement through maker-assisted selection in this family. At four of the five significant QTL, both alleles from the father and the mother showed significant effects, suggesting that QTL alleles of both parents are segregating. Whereas, at one significant QTL (*qBW3*), alleles from the father showed significant effects, but alleles from mother did not show effects, indicating the segregation of QTL alleles of the father, and fixation of QTL alleles from the mother. QTL allele substitution effects were also seen at suggestive QTL, suggesting the segregation of QTL alleles in both parents or one of the two parents.

Candidate gene approaches have been used for identification of QTL [45]. In a previous study, we demonstrated that the polymorphism in the gene *PVALB1 *located on LG23 was significantly associated with traits body weight and length in Asian seabass [30]. However, this gene was not polymorphic in the full-sib family for QTL analysis, and no significant QTL for traits was detected on LG23. Another potential candidate gene is the growth hormone (*GH*) gene, which was mapped near the microsatellite marker *Lca226 *on the LG3 in the first generation linkage map of Asian seabass [28]. This gene plays an important role in the growth of organisms. Previous studies in pig, chicken and cattle indicated that polymorphisms in the *GH *gene were associated with growth-related traits and other economically important traits [46-48]. Unfortunately, the microsatellite marker *Lca003 *located in the promotor of the *GH *gene was not informative in our mapping family. A significant QTL affecting body weight, explaining 8.8% of the phenotypic variance was mapped on LG3, but the position was about 13.9 cM away from the *GH *gene, suggesting the *GH *gene is not associated with growth-related traits in this family. To examine the effects of the *GH *and *PVALB1 *genes on economically important traits, more polymorphisms in these two genes will be detected.

## Conclusion

We detected five significant and 27 suggestive QTL for growth-related traits in an F1 family from a breeding stock of Asian seabass. This study presents the first example of QTL detection in an F1 family of a marine foodfish. The results presented will enable fine-mapping of the identified QTL for marker-assisted selection and eventually identifying individual genes responsible for these traits for direct selection

## Methods

### Source of fish

A broodstock consisting of 94 brooders from the Marine Aquaculture Center, Singapore were genotyped with nine polymorphic microsatellites (*Lca08*, *Lca20*, *Lca21*, *Lca58*, *Lca64*, *Lca69*, *Lca70*, *Lca74 *and *Lca98*) as described previously [6,28]. Genetic relationships among individuals were estimated by using genetic similarity according to Nei and Li [49]. Crosses were performed by selecting distantly related males and females to produce the largest genetic variation in the next generation. All brooders were tagged with passive integrated transponder (PIT) tags (IER, BP, France). From a mass-spawning of ten male and ten female brooders, which showed high genetic difference at the nine loci, about 20,000 fertilized eggs were brought to hatchery and were cultured in a one-ton tank following standard culture protocols [50] with modification until 30 dph. As Asian seabass juveniles are highly carnivorous, grading is necessary. From 30 dph on, these larvae were subsequently graded into three distinct sizes (large, medium and small) every week up to 65 dph, and transferred into cylindrical three-ton capacity tanks with continuous flow-through of optimum quantity of sand-filtered seawater. Juveniles were raised communally in their respective tanks with same stocking density, and were fed to satiation three times (9:30 am, 1:30 pm and 5:30 pm) daily on stringently sized pellets (INVE, PHICHIT, Thailand) until the experiment ended at 90 dph. Other conditions (i.e. air flow, water flow and light intensity) in the tanks were maintained as same as possible.

### Traits and DNA samples

At 90 dph, body weight (BW), total length (TL) and standard length (SL) of 600 progeny randomly collected from tanks with the largest and smallest fish were measured. Fulton's coefficients of condition (KTL and KSL) were calculated for each fish according to BW with TL or SL as described [51]. Fin clips of the parents and their progeny were collected and kept in absolute ethanol. DNA was isolated from fin clips using a new method developed by us [52]. The quality of DNA was checked on 1% agarose gel, and the quantity measured using Nanodrop (NanoDrop, DE, USA). DNA concentration of each fish was adjusted to 2.5 ng/μl and arrayed into 96-well PCR plates for later use. By genotyping the fish with nine microsatellite markers [6], pedigree was reconstructed. One parental pair producing the most offspring, 380 of them were used for linkage and QTL analysis.

### Microsatellites and genotyping

Ninety-seven microsatellite markers almost evenly covering the 24 LGs were selected from a first-generation linkage map of Asian seabass [28]. Primers were designed for each unique microsatellite using PrimerSelect (Dnastar, WI, USA). One primer of each pair was labeled with FAM or HEX fluorescent dyes at the 5'end. The PCR program for microsatellite amplifications on PTC-100 PCR machines (MJ Research, CA, USA) consisted of the following steps: 94°C for 2 min followed by 37 cycles of 94°C for 30 s, 55°C for 30 s and 72°C for 45 s, then a final step of 72°C for 5 min. Each PCR reaction consisted of 1× PCR buffer (Finnzymes, Espoo, Finland) with 1.5 mM MgCl_2_, 200 nM of each PCR primer, 50 μM of each dNTP, 10 ng genomic DNA and one unit of DNA-polymerase (Finnzymes, Espoo, Finland). Products were analyzed using a DNA sequencer ABI3730xl, and fragment sizes were determined against the size standard ROX-500 (Applied Biosystems, CA, USA) with software GeneMapper V3.5 (Applied Biosystems, CA, USA) as described previously[28].

### Linkage mapping

Linkage analyses were conducted through a series of pairwise comparisons between loci using software LINKMFEX [53]. The analyses were executed using segregation data for 97 microsatellite markers in 380 progeny. Pairwise recombination estimates obtained with module LINKMFEX were used as input into module MAPORD to determine linear assignments of markers within a linkage group. A minimum LOD score of 3.0 was used to assign markers to LGs. Map distances were calculated using the Kosambi function with module MAPDIS. Using module MERGE, new LG orders were built from pre-existing LG orders that were obtained from the male and female parents. Linkage map was constructed by averaging recombination rates across the sexes. Map graphics were drawn with MapMaker software [54].

### QTL analysis

With genotype data of the 97 markers and phenotypic data of the 380 progeny, QTL analysis was carried out using the program MapQTL 4.0 [25]. Interval mapping and multiple QTL model (MQM) mapping were utilized to detect any significance associating growth-related traits and marker loci in the data sets. The LOD score significance thresholds were calculated by permutation tests in MapQTL 4.0, with a genome-wide significance level of α < 0.05, n = 1000 for significant linkages and a linkage-group-wide significance level of α < 0.05, n = 1000 for suggestive linkages.

To determine the differences among the genotypes of markers nearest to each QTL, an analysis of variance (ANOVA), based on the genotypes (m1f1, m1f2, m2f1 and m2f2) of the marker locus lying closest to the peak in each of the QTL-containing genomic regions were also carried out to characterize the QTL allele substitution effects. A simple Bonferroni correction, to reduce the chance of type 1 error to 0.01 across all tests, gave a significance threshold of 0.0025, since we consider that tests were performed among four genotypes of each marker. This was conducted by using the general linear model (GLM) procedure of SAS (SAS Institute) and the Bonferroni method of multiple comparisons with α < 0.01. For the marker nearest to each QTL, effects of alleles within two parents and the interaction between paternal and maternal alleles were analyzed through ANOVA using GLM of SAS at α < 0.05.

## Abbreviations

QTL-quantitative trait loci; LG-linkage group; BW-body weight; TL-total length; SL-standard length; *qBW2-a*-QTL for body weight located on position a on linkage group 2; *qTL2-a*-QTL for total length located on position a on linkage group 2; *qBL2-a*-QTL for body length located on position a on linkage group 2; KTL and KSL: condition factors calculated based on the total length and standard length; cM-centimorgan; *GH*-growth hormone gene; ANOVA-Analysis of variance; and PVE-percentage of the phenotypic variance explained.

## Authors' contributions

GHY designed and started the project, and determined the final version of the manuscript. CMW designed the experiment, selected markers, conducted genotyping and QTL analysis, as well as drafted the manuscript. LCL measured and recoded the trait values and conducted most of genotyping. ZYZ helped in data collection, DNA extraction and genotyping. All authors have read and approved the final version of the manuscript.

## Figures and Tables

**Table 1:** Phenotypic values of growth-related traits of Asian seabass at 90 dph

Traits	Mean ± SD
Body weight (g)	29.4 ± 13.5
Total length (mm)	122.6 ± 20.1
Standard length (mm)	101.3 ± 16.8
KTL	1.49 ± 0.12
KSL	2.64 ± 0.22

KTL and KSL: condition factors calculated according to body weight (BW) and total length (TL)/standard length (SL).

**Table 2:** Location of QTL and magnitude of QTL effects

Trait	QTL name	LG	Nearest marker	LOD	LOD threshold		PVE (%)	Phenotype means				
							
					Genome-wide	Linkage-group-wide		m1f1	m1f2	m2f1	m2f2	Significance
Body weight (g)	*qBW2-a*	2	*Lca287*	5.25*	4.5	3.4	28.8	27.0	23.3	39.1	27.0	†‡
	*qBW2-b*	2	*Lca371*	4.62*	4.5	3.4	6.4	25.7	29.0	28.9	34.0	†‡
	*qBW3*	3	*Lca154*	5.33*	4.5	2.3	8.8	30.7	26.6	34.6	26.4	‡
	*qBW12*	12	*Lca178*	3.34	4.5	3.0	18.7	23.4	34.2	25.5	34.1	‡
	*qBW16*	16	*LcaE22*	1.96	4.5	1.7	2.5	28.5	26.9	30.9	31.3	†
	*qBW20*	20	*Lca234*	3.16	4.5	3.0	3.8	32.8	26.4	29.6	29.7	‡§

Total length (mm)	*qTL1*	1	*Lca148*	4.65	6.1	4.4	8.8	134.8	111.7	106.8	135.8	§
	*qTL2-a*	2	*Lca287*	6.59*	6.1	5.4	58.9	119.7	104.2	138.5	131.5	†‡
	*qTL2-b*	2	*Lca371*	5.45	6.1	5.4	7.6	117.1	123.2	122.9	130.9	†‡
	*qTL3*	3	*Lca154*	5.08	6.1	2.3	8.3	125.9	119.1	131.0	119.4	‡
	*qTL12*	12	*Lca178*	3.26	6.1	3.3	22.6	113.8	131.6	116.7	131.4	‡
	*qTL16*	16	*LcaE22*	2.06	6.1	1.7	2.6	121.5	120.3	126.5	126.2	†

Standard length (mm)	*qSL1*	1	*Lca148*	4.65	6.1	4.5	8.3	111.4	92.3	88.3	112.3	§
	*qSL2-a*	2	*Lca287*	6.30*	6.1	5.4	59.7	98.3	86.1	114.7	108.9	†‡
	*qSL2-b*	2	*Lca371*	5.05	6.1	5.4	7.2	99.4	100.9	99.0	108.4	†‡
	*qSL3*	3	*Lca154*	5.16	6.1	2.3	8.4	104.1	98.3	108.4	98.7	‡
	*qSL12*	12	*Lca178*	3.32	6.1	3.2	21.9	93.7	109.2	97.0	108.1	‡
	*qSL16*	16	*LcaE22*	2.19	6.1	1.7	2.7	100.4	99.3	104.7	104.3	†

KTL	*qKTL6-a*	6	*Lca409*	2.76	4.6	2.6	8.1	1.51	1.50	1.43	1.50	†‡
	*qKTL6-b*	6	*Lca223*	3.99	4.6	2.6	10.7	1.46	1.45	1.49	1.55	†
	*qKTL7*	7	*Lca436*	3.55	4.6	2.3	4.3	1.50	1.52	1.46	1.46	†
	*qKTL11-a*	11	*Lca315*	2.48	4.6	2.4	6.7	1.46	1.45	1.52	1.51	†
	*qKTL11-b*	11	*Lca058*	2.43	4.6	2.4	5.9	1.51	1.51	1.45	1.45	†
	*qKTL12*	12	*Lca115*	2.87	4.6	2.8	18.3	1.50	1.44	1.44	1.55	†‡
	*qKTL16*	16	*LcaE22*	1.99	4.6	1.7	2.5	1.51	1.48	1.46	1.48	†
	*qKTL21*	21	*Lca349*	3.78	4.6	2.3	4.9	1.53	1.47	1.47	1.46	‡

KSL	*qKSL6-a*	6	*Lca409*	2.38	6.1	2.4	8	2.67	2.66	2.53	2.67	†‡
	*qKSL6-b*	6	*Lca223*	3.22	6.1	2.4	3.8	2.59	2.60	2.64	2.70	†
	*qKSL7*	7	*Lca436*	3.88	6.1	2.8	4.6	2.68	2.67	2.58	2.58	†
	*qKSL16*	16	*LcaE22*	2.29	6.1	1.7	2.9	2.67	2.63	2.57	2.63	†
	*qKSL21-a*	21	*Lca349*	3.26	6.1	2.4	4.2	2.69	2.65	2.58	2.59	†
	*qKSL21-b*	21	*Lca421*	3.24	6.1	2.4	6	2.63	2.71	2.56	2.61	†‡

For each QTL detected, the linkage group maximum LOD score, and percentage of the phenotypic variance explained (PVE) are indicated. The genome-wide LOD significance thresholds are 4.5 body weight, and 6.1 for total length and body length. Mean phenotypic values of each trait were also calculated for those progeny with the alternate alleles at the most closely linked microsatellite markers, inherited from the male parent (m1 or m2), alleles inherited from the female parent (f1 or f2). *: Significant QTL; †: Significant effect within male alleles, ‡: Significant effect within female alleles; and § Significant interaction between alleles from male and female.
